# Actions taken to safeguard the intended health care chain of older people with multiple diagnoses - a critical incident study

**DOI:** 10.1186/s12912-022-01039-1

**Published:** 2022-09-21

**Authors:** Lena-Karin Gustafsson, Viktoria Zander, Anna Bondesson, Tina Pettersson, El-Marie Anbacken, Gunnel Östlund

**Affiliations:** 1 Division of Caring Science, School of Health, Care and Social Welfare, Mälardalens University, Box 325, 63105 Eskilstuna, Sweden; 2 Division of Physiotherapy, School of Health, Care and Social Welfare, Division of Physiotherapy, Mälardalens University, Västerås, Sweden; 3 Division of Social work, School of Health, Care and Social Welfare, Mälardalens University, Eskilstuna, Sweden

**Keywords:** Critical incidence technique, Home-based healthcare, Multiple diagnoses, Older people, Person-centred care, Care-coordination

## Abstract

**Background:**

Older people with multiple diagnoses often have problems coping with their daily lives at home because of lack of coordination between various parts of the healthcare chain during the transit from hospital care to the home. To provide good care to those persons who have the most complex needs, regions and municipalities must work together. It is of importance to develop further empirical knowledge in relation to older persons with multiple diagnoses to illuminate possible obstacles to person-centred care during the transition between healthcare institutions and the persons livelihood. The aim of the present study was to describe nurses’ experienced critical incidents in different parts of the intended healthcare chain of older people with multiple diagnoses.

**Methods:**

The sample consisted of 18 RNs in different parts of the healthcare system involved in the care of older people with multiple diagnoses. Data were collected by semi structured interviews and analysed according to Critical Incident Technique (CIT). A total of 169 critical incidents were identified describing experiences in recently experienced situations.

**Results:**

The result showed that organizational restrictions in providing care and limitations in collaboration were the main areas of experienced critical incidents. Actions took place due to the lack of preventive actions for care, difficulties in upholding patients’ legal rights to participation in care, deficiencies in cooperation between organizations as well as ambiguous responsibilities and roles. The RNs experienced critical incidents that required moral actions to ensure continued person-centred nursing and provide evidence-based care. Both types of critical incidents required sole responsibility from the nurse. The RNs acted due to ethics, ‘walking the extra mile’, searching for person-centred information, and finding out own knowledge barriers.

**Conclusions:**

In conclusion and based on this critical incident study, home-based healthcare of older people with multiple diagnoses requires a nurse that is prepared to take personal and moral responsibility to ensure person-centred home-based healthcare. Furthermore, the development of in-between adjustments of organizations to secure cooperation, and transference of person-centred knowledge is needed.

## Introduction

Society in most countries around the world places increased demands on healthcare providers, including county councils and municipalities, to carry out activities to handle and secure health care needs jointly for older people with multiple diagnoses. The Swedish government appointed a national ‘elderly coordinator’ to focus on the most ill older people in January 2011. The report “Five years focusing on the most ill elderly” 2011–2015 [1], provides a description at a regional level which has been interpreted to a national level of how to care for the older population extending their time living at home. The older people referred to here as being most ill, having complex needs are defined as having three or more chronic conditions affecting their capacity to live a ‘normal’ life [2]. The welfare goal has been for everyone to be able to age with security and self-determination, with access to good care and home assistance through strengthening and intensifying the collaboration between different healthcare organizations managed by the regions and municipalities. In Sweden the responsibility of healthcare is owned by the regional county councils and for older persons living at home the municipality at a local level is responsible for health and social services such as home help and daily meals if needed. The National Board of Health therefore proposed the legislation [3] that requires regions and municipalities to carry out health care and rehabilitation activities jointly for older people with extensive needs, so that the individual receives coherent care and social services [4]. All care and welfare services are charged and paid by the person to either the region or to the municipality depending on what services you need.

Most older people with multiple diagnoses need frequent hospital care even if home-based healthcare is delivered in the person’s housing. However, transferring an older person to an emergency department means there is a high risk of them developing further health complications [5]. Previous research shows, for example, that shorter hospital stays present a lower risk of both postoperative complications and infections compared with extended stays [6]. Discharge from hospital of these patients has been shown to be associated with multiple distress and further health decline unfortunately leading to hospital readmission [7–9]. Frail older people with cognitive impairment, who need frequent care, should and can be prevented from unnecessary hospitalisation based on the legalisation of the new act [3]. However, Swedish ambulance care receives repeated calls from older people living at home who are worried and ask for more advanced care [10], which might be avoided if there was extended planning of welfare services and healthcare for the older person [5] and more healthcare opportunities for older people living at home, including having a well structed healthcare chain in case of emergency and vulnerable needs.

The healthcare chain refers to how healthcare professionals from the different organizational residence through collaboration will deliver safe transition for the patient between hospital and primary healthcare [11] and home care in the person’s own accommodations. According to research, it is important that all partners are prepared to implement joint work processes in the discharge chain from a hospital visit. In job responsibilities and content RNs needs to complement each other to cover the various needs of the person with multiple diagnoses. A sensitive chain link that research has identified as offering fragmented care during the transit of older people from hospital care to the home, not least with the message that social needs seem to be as important as medical needs [12]. According to older persons’ perspectives, multi-professional collaboration is one success factor for satisfaction with the healthcare system [13]. However, this requires enhanced interprofessional communication and availability of shared documentation [14]. Likewise, Morris [15] called for extended coordination of care for example by shared IT systems between healthcare and social care organizations and this research study also asked for available geriatric competency, including knowledge and abilities among professionals to deliver care with dignity and kindness. Theoretically this study takes its departure in theory of person-cantered care as one of the nurse’s core competences described by Mc Cormack et al. [16]. The foundation of person-centred care is respect for the person, the person’s right to self-determination and equal understandings. Person-centred care is advocated where accurate interventions according to physical condition or health status are acknowledged and older adults’ own values are integrated. Jarling [17] points out that the complexities of care in the home often include relational and social aspects, which again underlines person-centred holistic caring especially when focusing on older people’s needs. Research has also identified next of kin responsibilities in keeping track of and communicating information between different caregivers to facilitate care for older persons. The older adult is expected to be an active partner in their own care but is often excluded from the information flow [18]. A common framework with organizationally based values that takes the person’s resources into consideration is crucial, especially to unveil experiences of vulnerability, dependence, and exposed situations in the discharge process of hospital care [19]. Mutual goals, common understanding and trust between the person and among caring professionals could therefore contribute to secure person-centred care [20]. emphasize that performance and results can be seen from different stakeholder perspectives where the assessment of what is acceptable care depends on each individual’s perspective. Each person’s difference in performance values must be made visible to create a common basis for what is acceptable and successful care. Lack of collaboration in the transition from hospital to home-based care contributes to difficulties in getting a common understanding and interferes with the possibility to provide a transition based on individual needs, values, and expectations [21]. It is therefore important that healthcare efforts are coordinated based on the needs and conditions of the older people with multiple diagnoses, in collaboration with their relatives [12, 15].

Informal caregivers carry a heavy responsibility for their relatives’ wellbeing and must collaborate with various professional service providers. The social services are experienced as fragmented and are often provided by many different service providers [22], sometimes leaving the person and relatives as uninformed agents in relation to care aspects [21]. Resent research, however, points to the need for multi-professional cooperation in health and welfare services, such as hospital clinics, home-based healthcare and social services, day centres and short-term respite in care homes [22]. Hansson et al., [23] point out several obstacles for effective communication and care planning for frail older people. Such as lack of communication with older persons and their relatives, lack of collaboration between professional caregivers and the lack of responsibility for care planning including resources not being distributed according to the actual needs of the older person. This research project was supported by the region and local municipality to develop further empirical knowledge in relation to the intended healthcare chain especially related to older persons with multiple diagnoses to illuminate possible obstacles to person-centred care during the transition between healthcare institutions and the persons livelihood. The purpose was to expand the body of knowledge and to receive more empirical evidence of nurses working situations in relation to older people’s person-centred care.

The aim of the present study was to describe nurses’ experienced critical incidents in different parts of the healthcare chain working with older people with multiple diagnoses.

## Materials & methods

### Study design and setting

The study had a qualitative descriptive design and was analysed according to Flanagan’s [24] Critical Incident Technique (CIT). The setting focused was on the intended healthcare chain for older people with multiple diagnoses.

### Participants and data collection

The inclusion criteria were RNs (registered nurses) responsible of caring for older people with multiple diagnoses and their movements between resources in municipal home healthcare (municipal nurses) towards regionally organized in/out-patient clinics to medical hospital care (regional nurses), see in detail Table 1. The two main health and welfare organizations (municipal and region) in one middle sized city in southern Sweden were informed about and invited to the project first orally in a presentation and then further by mail. Eighteen nurses answered the mail invitation and were contacted by the research team to receive more information about the research project and if the nurse chose to take part an interview, time was organized (demographic information of the sample is found in Table 1). The interviews took place in a separate and quiet room adjacent to the nurse’s workplace chosen by each participant. The interviews were recorded digitally and transcribed verbatim.

**Table 1:** Demographics RN CIT

*Background variables*	*n*
*Sex*	
Female	18
Male	-
*Age*	
23-30	2
31-40	1
41-50	6
51-60	9
*Experience in the profession (years)*	
3-5	3
6-10	1
>10	14
*Level of education* (specialists)	
District nurse (nurse with further education for outpatient health care)	6
Other specialized RN education	1

### Critical incidence technique

The semi-structured interviews were conducted with Critical incident technique (CIT) which contains open questions that encourage storytelling about critical situations at work. In line with CIT, the respondents were given the opportunity to describe both positive and negative critical incidents that they perceived as being important. For example, −can you tell me about a situation with desirable conditions for a safe and sustainable situation. Or -can you tell me about a situation with opposed to desirable conditions in the home for older people with multiple diagnoses? CIT has been used by several health researchers for example Kontio et al., [25] Östlund, et al., [26] and Kyndi Pedersen et al. [27], critical incidents, so called problematic situations that have occurred in the participant’s (work) life, are explored by the interviewer. The use of CIT involves a focus on human actions in recently experienced situations, as opposed to investigating what happened in past (work) life.

### Analysis

The interviews were transcribed, analysed, interpreted, and categorized according to the Critical Incident Technique in Flanagan’s [24] spirit in detail described by Fridlund et al. [28], with focus on consequences and actions for people. The analysis of data started with vertical reading of each interview to become familiar with their content. As quality control to ensure the trustworthiness of the analysis, the reading took place independently with four of the authors involved. The selected CIT’s with experienced consequences and actions related to the aim of the study were compared, delimited, and put into horizontal analysis tables. A total of 169 critical incidents were identified in this phase. A comparison of these tabled incidents was then made to find similarities and differences categorizing them together in 13 sub-categories and five categories, with four main areas describing experienced consequences. Fifty actions were identified, with eight sub-categories and four categories, and two main areas describing actions to recent situations. Here, the purpose of a category is to describe the character of the sub-categories found, while the purpose of the main areas is to describe the overall results of CIT analysis.

## Results

The result showed the main areas of experienced critical incidents as being organizational restrictions in providing care and limitations in collaboration. Actions took place due to the lack of preventive actions for care, difficulties in upholding patients’ legal rights to participation in care, deficiencies in cooperation between organizations as well as ambiguous responsibilities and roles. Critical incidents that required moral actions to ensure continued person-centred nursing and provide evidence-based care were also experienced. Both types of critical incidents required sole responsibility from the nurse. The RNs acted due to ethics, searching for person-centred information, and finding out own knowledge barriers (see Table 2).

**Table 2:** Summary of the main areas of critical incidents, categories and sub-categories describing experienced consequences in recent situations. Number of critical incidents in brackets

Main area	Categories	Sub-Categories
**Organizational restrictions in providing care**	Lack of preventive circumstances for care	Regulations prevent the performance of certain medical treatment at home, even though there is competence (8)
		Lack of access to information due to various computer systems (4) Limited opportunities for home visits (5)
	Patients’ legal right to decisions	Individual’s self-determination challenges care (29) Relatives have opinions about care (3)
**Limitations in collaboration in healthcare**	Deficiencies in cooperation	Repeated hospital visits (5) No support from HC (14) Lack of collaboration between professions (18)
	Deficiencies in communication	Opportunity for direct contact with other healthcare institutions shortcomings (9) Over-reporting between different care agencies (22)
	No principal caregiver	Nobody wants to take responsibility (10) Unclear assignments who is responsible between professions (8) Too many actors around each patient (34)

### Main area - organizational restrictions in providing care

#### Lack of preventive circumstances for care

According to the interviews, regulations prevented the municipal RNs’ performance of certain medical treatments at home, even though professional competence was available. Experiences of critical incidents due to similar non-preventive regulations led to frustration and sometimes to an attitude of “let go”. For example, RNs often wanted, but were not allowed to, administer an intravenous drip in the home due to regulations. The absence of nurse mandate meant that the person in their own dwelling needed to be sent to the emergency room instead of benefitting from treatment from the nurse already present in their home.

I felt as a nurse that I just wanted to help him. But there was so much more. Rules and here and there, and what you could do at home, and then it would be antibiotics intravenously, and we were not allowed to do that. (Municipal home health care nurse).

Lack of access to information due to various computer systems was also an experienced dilemma for the RNs s in different organizations. The RNs employed by the municipality could read certain parts but not write in the hospital’s computer documentation system. Regional RNs in hospital care, however, did not have access to the municipal computer documentation system, which included all notes taken by the caregivers working within social services who had most contact with the older person in their home.

And it is hard since we don’t have the same …. we don’t have the same computer system … so we cannot get access and read about how this patient is doing. And since the patient doesn’t visit the reception any longer we don’t even get information within the region. So, there we are in between in a way. (Regional nurse at the emergency ward).

Limited opportunities for home visits were experienced as critical incidents for the RNs employed by the municipality, since they often knew the patient better and recognised that a possible home visit by a physician could prevent hospital admission.

I also tried to get a physician out, or home visit. But then we thought... - what could the primary healthcare centre physician really do? It does not feel okay to send a person who has a cancer tumour that cannot be operated on to the emergency clinic. (Region nurse at the primary health care center).

RNs were often frustrated in the knowledge that they were acting incorrectly and not offering person-centred care, especially when this involved palliative (end of life) care in the person’s home. RNs often found they were forced to send the person to hospital as there were no other options since physicians are not allowed to attend home visits in Sweden.

### Patients legal right to decisions

According to the RNs, the patient’s legal self-determination rights could stand in the way of providing “sufficient” professional care. Patients’ rights were sometimes experienced as obstacles although all RNs recognised the importance of complying with them. For example, one patient did not allow the nurse to contact other care providers for advice or to read the medical records.

I have a patient who has had multiple diagnoses for a long time. This is difficult because [the person] does not allow us to read the medical records and does not allow us to contact other caregivers until [the person] tells us to. The patient is an obstacle to optimal care in this case. (Municipal home health care nurse).

In addition, according to the RNs some relatives had too many opinions about care and that was experienced as critical incidents in their work. Such as when the relative wanted to stay in charge and control the care of their next of kin. *“Relatives are the biggest resource and asset we have, but sometimes it can be very complicated when they are poking around everywhere, controlling”.* (Municipal home health care nurse).

When planning for discharge from hospital occasionally the RNs experienced so much pressure from dissatisfied relatives that it almost affected the quality of planned care as well as interfered with the patients’ trust in the planned care. “*And then you have the pressure from relatives, and when they are dissatisfied. The patient is not so happy …*” . (Regional nurse at medical care ward).

The RNs expressed an insight that both the patient and their relatives would and should obviously be involved and participating in person-centred care throughout the health care chain. Still there was a dilemma to work the discharge out in the best interests of the patient.

### Main area - limitations in collaboration in healthcare

#### Deficiencies in cooperation

Repeated hospital visits for persons with multiple diagnoses were critical incidents brought up by the RNs in different parts of the health care chain. Repeated hospital visits are for the RNs well-known obstacles in caring for older people with multiple diagnoses. The RNs suggested that a mobile team could work closely with them instead. This team would include cooperation with a physician. This was presented as a possible way to handle the lack of cooperation and trust that existed at present and as a result keep the person in their own home. “*I experience that in home-based healthcare … It’s like … You send in our care recipients more [to hospital]. That’s how it is. We do not keep them at home for long. Needed resources do not exist today”.* (Municipal home healthcare nurse) Referring to the absence of a physician that could make the decision.

In addition, RNs experienced critical incidents due to having no or limited support from Primary health care centres, which were supposed to be the home-based healthcare RNs’ collaborative partner in the care of patients in their home. Many municipal home healthcare nurses felt abandoned and lonely in their caring work due to this lack of cooperation around the patients’ health and care.

In home-based healthcare, we had a woman who had uncontrollable diabetes, and her blood sugar-level fluctuated, so we had huge problems. The home healthcare personnel called us all the time. So, we spent a lot of time contacting the Primary healthcare centre, but they ignored us, usually we got no response at all. (Municipal home healthcare nurse).

The RNs in the municipal home healthcare also described critical incidents due to an experienced lack of collaboration between RNs from different organisations and between different professions. Lack of cooperation among professions sometimes originated in hierarchies in the healthcare organizations or in the different approaches of the different professions’ practical and theoretical traditions.

What goes under the responsibility of the home-based healthcare, the nurses, what can they do, what can they do and what goes under our responsibility? That’s the hard part. And it seems as if no one knows the boundary. No. So the cooperation isn’t exactly optimal. (Regional nurse in a primary health care center).

### Deficiencies in communication

The RNs experienced that a lot of their time was taken up trying to contact other healthcare institutions, these were also critical incidents in nursing work. Their attempts to make a connection often failed partly due to lack of adjusted digital technique …” *If I am with a patient, I don’t have a computer. I have a telephone. About getting in touch with someone, no, then I have to send a message … “*(Municipal home healthcare nurse).

The interviewed RNs revealed critical incidents that led to a lack of communication and in some cases a lack of reporting between different care agencies trying to find out what had been said and done regarding one patient. The coordination nurse at the primary healthcare centre used to prioritize and handle the contact with relatives if a patient was going to the primary healthcare centre, thus avoiding a gap in communication. However, the home-based healthcare nurse sometimes became involved with the patients and their relatives although the patients were not sufficiently enrolled. Therefore, they did not always have insight into what was said and done regarding the needed care via the primary healthcare centre.

So, there I was in between. I couldn’t enrol her to home-based healthcare because there were no injections, no palliative care … She lived at home, she felt a bit nauseous, but she wanted to take care of her medicines by herself. (Nurse at the primary healthcare centre).

### No principal caregiver

The RNs experienced that nobody really wanted to take overall responsibility. The interviewed RNs identified unclear assignments and ambiguous roles. Responsibility was unclear between different organizations, there is no principal caregiver leading to loss of responsibility or competing responsibilities. Critical incidents were also that the RNs lacked the mandate to give directives to those employed in other organizations.

I have unfortunately experienced that it is a bit difficult because we have different principals, that it can sometimes be a bit … That I who come from inpatient care do not really have the mandate to require the home-based healthcare service and ask them for help // We must go in the right direction and to the right organization. Yes, but now it's Friday afternoon, the patient is in front of us. What are we going to do about the situation? (Nurse emergency ward)

In addition, RNs experienced critical incidents due to lack of clear responsibility and because there were too many actors around each patient. It was difficult to know who was in charge and what they oversaw if there were many caregivers. For example, the primary care clinics within the region did not know that the municipality had responsibility for the patient’s medication. Thus, when the region gave the patient medication without notice, the patient became incorrectly medicated.

It became very unfortunate considering that the cardiology department and all other clinics did not know that I was responsible for the medications. So, they changed them all the time. So, he has probably never had such wrong medication as under their responsibility. (Municipal home healthcare nurse)

Table 3 shows a summary of the main areas, categories and sub-categories describing actions in recent situations with a focus on how to cope with the critical incidents.

**Table 3:** Summary of the main areas of critical incidents, categories describing actions in recent situations. Numbers of actions in brackets

Main area	Categories	Sub-Categories
**Moral actions by the RNs in nursing**	Acting due to nursing ethics	The nurse takes her own moral responsibility (13) The nurses felt responsibility to act (3)
**Providing evident care**	Walking the extra mile	Stretched the boundaries (4) Doing the extra (5)
	Actively searching for information	The nurse herself actively searches for information (10) Engage relatives (6)
	Acting for security	Knowing boundaries (3) Convey safety (6)

### Main area- Moral actions by the RNs in nursing

#### Acting due to nursing ethics

The RNs often took their own moral responsibility due to their ethos as nurses. Their actions were determined by a responsibility towards both relatives and the older adult with multiple diagnoses in a vulnerable situation.

*…* a complicated matter when he was going home … You almost had to reinvent the wheel, because no one knew of any regulations about whether you can have self-care on certain things? And can you go home and leave certain things to a wife who is over 80 years old? (Regional nurse at medical care ward)

The RNs felt their responsibility to act and advocate due to the patients’ health and wellbeing. Even to monitor the patient’s right to survival. The RNs themselves sometimes increased the number of home visits and tried to influence the scheduling of visits to reduce the number of visits from other healthcare professionals to safeguard these vulnerable patients.

I had wanted to have a proper care plan, for me to know as well. It felt like things were left to go as they pleased all the time. And that I helped without really having anyone … I had no assignment to help him. But I had to help him to survive... (Municipal home healthcare nurse)

#### Walking the extra mile

Some of the critical incidents and the expressed actions showed that the nurse’s altruism stretched the boundaries of what they had needed to do according to their own moral duties. The RNs did extra visits and helped the patient as much as they could, because they understood that the patient needed it. These nurses did everything needed so that it would work out for the patient at home. For example, the nurses rescheduled their working day, rescheduled other commitments, or looked in between other visits to check up.

We have really done our utmost for the patient … You see the patient in this. Because the patient is in his home 24 hours seven days a week and really makes sure it works. You may be trying to change your schedules. One understands that there is an individual there on the other side. (Nurse at the primary healthcare centre)

### Main area- Providing evidence-based care

#### Actively searching for information

Working for a sustainable situation at home for older people with multiple diagnoses included actively searching for the health information the nurse needed to safeguard the person. This situation differed from other nursing contexts where there used to be both colleagues and physicians to ask when solving a health problem. Sometimes the municipal RNs’ working situations were experienced as being very lonely, and it required being sensitive when working with vulnerable older people with multiple diagnoses that might demand specific solutions in every case, situations the RNs may not have experienced before. “*It is energy-intensive, time-consuming and unsafe from a patient safety perspective when the nurse herself must search for the health information in the correct place.”* (Municipal home healthcare nurse).

The municipal RNs actively engaged relatives as an important source of information for the RNs to provide proper evidence-based care. The nurse also worked a lot to engage the relatives in the care, providing them with information of planned care and evidence around the patient’s conditions.

#### Acting for security

Knowing the boundaries is an aspect or precondition for conveying security and means knowing when to hand over severe health difficulties that RNs are not able to handle by themselves. Even though the ambition was to make the person as secure as possible and provide care in their own home, the RNs knew the boundary of what they could manage and when to hand over responsibility to other more specialized caregivers or physicians. The RNs had an insight into how far they could provide evidence-based care in a patient’s home.

I did not have her enrolled, but I said to her daughter "of course I can come home and make an assessment if you wish, but based on your description, I think she should go to the hospital, so it does not spread or something else in the stomach” (Municipal home healthcare nurse)

After all, actions to convey safety were experienced as crucial everywhere in the intended healthcare chain working for a safe and sustainable situation at home for the older people with multiple diagnoses in home-based healthcare. “*… If a person is safe and gets the help or information at home, that person does not go back and forth to the hospital, then it feels okay.”* (Municipal home healthcare nurse).

## Discussion

The present study focuses on critical incidents experienced by RNs in different parts of the intended healthcare chain working for and responsible of a safe and sustainable situation in the home for older people with multiple diagnoses. The interviewed RNs identified 169 critical incidents primarily related to the RNs’ experiences of organizational restrictions in providing person-centred care related to lack of care communication between nurses from the different organizations. The experiences of mixed work responsibilities or not knowing who the main responsible organization is made nursing difficult and less motivating, since there was not enough trust between the different organizations. This limited collaboration between the healthcare organizations made communication efforts even worse because you might also have to increase your efforts to understand what was decided on a particular patient. The experienced difficulties in cooperative care between regional and municipal healthcare organizations could be compared to the care chain fragmentation described by Neiterman et al., [12]. The use of different web platforms meant that communication was impossible and important medical records could not be read. Likewise, Lundborg et al., [14] show the importance of documentation and communication to achieve collaboration among professionals. Morris [15] also identified the need for shared IT systems almost 10 years ago. There are differences in accessing information between nurses in home-based healthcare and institutional regional care where nurses have access to medical journals. In home-based healthcare you must make use of the different professions and relatives to receive enough information on the person, and the person’s relational and social aspects are important pieces of knowledge when delivering person-centred, safe, and sustainable care [16, 17].

Two main areas of actions were used by the municipal RNs to handle the experienced critical incidents. RNs took personal responsibility to care for the person to provide evidence-based care without the support from colleagues. The municipal nurse had to accordingly keep the person safe and ensure person-centred care in the home environment was provided and to find out what health information and treatment was evident for each patient. RNs in community care also had to decide on their own when to ask for in-patient care, often feeling lonely in their responsibility. However, nurses employed by the region felt they had more support in their decisions, from, for example, physicians. Being solely responsible without having enough health information, or lacking professional cooperation goes beyond what research points out as being needed in the care of older people [14, 22]. Older people with multiple diagnoses often have problems coping with their daily lives because of a lack of coordination and coordination between the various parts and actors of the care. To provide good care to those patients who have the most extensive needs, regions, and municipalities need to work together with direct and daily contact. The voiced problems by municipal RNs in the present study regarding the lack of coordination of medication between different healthcare organizations is a particularly urgent problem. The healthcare and welfare services that the older persons today have access to in their own homes lack coordination, which means that the person might seek medical care from different healthcare institutions and organizations on their own and in the worst-case scenarios without the different healthcare professionals knowing about the person’s previous health conditions or retrieved medication. The municipal RNs in the present study experienced a great number of critical incidents related to a lack of communication between care professionals but also in interactions with next of kin and the patient.

In Sweden, as in other Western countries there is a massive shift from hospital centred care to home-based healthcare [1]. Changing work practices is difficult, but it is important that different principals that provide healthcare along with their employed healthcare and welfare professionals are prepared to implement joint work processes in the discharge chain [7–9]. These systems must provide routines that safeguard evidence-based care that does not have to rely on the RNs’ personal moral feeling of responsibility as the case is today and shown in this study.

At present, older people in Sweden can ask for or rather require care in their home, even if they have comprehensive care needs and suffer from multiple diagnoses [2]. However, older people with multiple diagnoses are unfortunately frequently sent back and forth in the healthcare system [10] This is sometimes based on their own choices and other times due to a lack of communication between healthcare professionals, as the present study has identified. This group of older people also meet many different healthcare professionals in the various care settings during one ordinary month often without them questioning the lack of continuity [3]. The extended patient rights to participation in care along with the increasing numbers of older people in Western societies where not everyone can be cared for in hospitals [17] requires new knowledge.

### Limitations of the study

It is always critical to capitalize on the respondents’ own stories, thus avoiding the loss of information that can occur when complex narratives are reduced to simple descriptive categories [29]. The direct quotes from the interviews strengthen the accuracy of the study, although it is also critical to focus on the respondents’ own stories, their actual way of telling them, while at the same time trying to minimize the subjectivity that is always a risk in qualitative research [30]. We believe that the multidisciplinary research group to improve the trustworthiness of the analysis. The categorizing of incidents into sub-categories was conducted in cooperation with four of the members of the research team, experienced both in theory and the context of the healthcare chain of older persons with multiple diagnoses. Furthermore, the validity of the findings was supported by all researchers in the project group. This consisted of six researchers representing the four different professional backgrounds of social workers, district/home-based healthcare RNs, psychiatric nurses and physiotherapists, as well as the different scientific fields of physiotherapy, social work, psychiatric care, and primary-home-based healthcare.

## Conclusions

This critical incident study identifies the challenges municipal RNs meet when trying to uphold sustainable person-centred care in the home for older people with multiple diagnoses. The intended healthcare chain is unstructured and lack enough unity, moreover RNs with sole responsibilities doesn’t secure evidence-based care. Similar challenges seem to occur in most countries, when trying to carry out joint activities. Therefore, this cannot be considered a Swedish challenge but something for healthcare providers to deal with around the world. RNs are currently expected to take personal and moral responsibility to secure evidence-based, person-centred, and safe home-based healthcare. Lack of communication and coordination between different healthcare providers and organizations give older people, especially with multiple diagnoses, an insecure healthcare situation.

## Data Availability

The datasets generated and/or analysed during the current study are not publicly available due to limitations of ethical approval involving the patient data and anonymity but are available from the corresponding author on reasonable request.

## Abbreviations

IT systems: Information technology systems
CIT: Critical Incident Technique
RNs: Registered nurses
D. nr: Diarie nummer/Eng. case reference number
DPA: The Swedish Data Protection Authority
GDPR: General Data Protection Regulation
